# Functional electrical stimulation of gluteus medius reduces the medial joint reaction force of the knee during level walking

**DOI:** 10.1186/s13075-016-1155-2

**Published:** 2016-11-03

**Authors:** Lance Rane, Anthony Michael James Bull

**Affiliations:** Department of Bioengineering, Imperial College London, Bessemer Building, South Kensington Campus, London, SW7 2AZ UK

**Keywords:** Osteoarthritis treatment, Medial knee joint reaction force, Functional electrical stimulation, Gluteus medius, Musculoskeletal modelling

## Abstract

**Background:**

By altering muscular activation patterns, internal forces acting on the human body during dynamic activity may be manipulated. The magnitude of one of these forces, the medial knee joint reaction force (JRF), is associated with disease progression in patients with early osteoarthritis (OA), suggesting utility in its targeted reduction. Increased activation of gluteus medius has been suggested as a means to achieve this.

**Methods:**

Motion capture equipment and force plate transducers were used to obtain kinematic and kinetic data for 15 healthy subjects during level walking, with and without the application of functional electrical stimulation (FES) to gluteus medius. Musculoskeletal modelling was employed to determine the medial knee JRF during stance phase for each trial. A further computer simulation of increased gluteus medius activation was performed using data from normal walking trials by a manipulation of modelling parameters. Relationships between changes in the medial knee JRF, kinematics and ground reaction force were evaluated.

**Results:**

In simulations of increased gluteus medius activity, the total impulse of the medial knee JRF was reduced by 4.2 % (*p* = 0.003) compared to control. With real-world application of FES to the muscle, the magnitude of this reduction increased to 12.5 % (*p* < 0.001), with significant inter-subject variation. Across subjects, the magnitude of reduction correlated strongly with kinematic (*p* < 0.001) and kinetic (*p* < 0.001) correlates of gluteus medius activity.

**Conclusions:**

The results support a major role for gluteus medius in the protection of the knee for patients with OA, establishing the muscle’s central importance to effective therapeutic regimes. FES may be used to achieve increased activation in order to mitigate distal internal loads, and much of the benefit of this increase can be attributed to resulting changes in kinematic parameters and the ground reaction force. The utility of interventions targeting gluteus medius can be assessed in a relatively straightforward way by determination of the magnitude of reduction in pelvic drop, an easily accessed marker of aberrant loading at the knee.

## Background

There has been increasing recognition of a biomechanical basis for joint pathology in osteoarthritis (OA), and with this hope that a new generation of disease-modifying therapies might follow. Of particular significance is the emergence of aberrant joint loading as driver of disease. In healthy individuals, the medial compartment of the tibiofemoral joint bears 2.5 times the load borne by the lateral compartment [1]; in patients, it is the usual site of manifestation of OA of the knee [2]. Once OA is established, the external adduction moment of the knee (EAM), a more readily determined correlate of the internally acting medial knee joint reaction force (JRF), has been shown to predict disease severity [3] and risk of progression [4], suggesting its utility as a clinical biomarker targeted for reduction.

When working in the clinical domain there is a clear need for accurate measures of internally acting forces, but studies have shown significant inter-individual variation in the relationship between the EAM and the medial knee JRF [5]. Previously, measurement of internal forces had been possible only through the use of instrumented internal prostheses [6, 7], but advancements in computational musculoskeletal modelling now enable their reliable determination non-invasively, facilitating wide-scale data collection. Musculoskeletal models perform inverse dynamics analysis within the context of a rigid body framework provided by the skeleton, where muscles act as force generators able to cause accelerations. Kinematic (joint angles and segment positions) and kinetic (ground reaction force (GRF)) data are used to formulate the equations of motion at each time step; solving these yields muscle, joint and ligament forces. The system is indeterminate - there are more solutions than there are equations - reflecting the large number of possible combinations of muscular activations that may result in a given movement. This necessitates the application of certain assumptions regarding muscular activations in a process known as optimisation, which models empirical ideas about how force generation might be shared optimally amongst muscles. In mathematical terms, optimisation generally involves finding solutions that minimise an *objective function* reflecting the total sum of some function of muscle stresses [8]. The mathematics has basis in physiology; the incorporation of muscle stress into the objective function results in force generation scaling roughly with muscle size, with the biggest muscles contributing most to the movement task. This means that individual muscle stresses are kept low and so their capacity for prolonged or repeated contraction high. Thus, as pointed out by Crowninshield and Brand [8], minimising the objective function is equivalent to maximising muscular endurance, and this feature of the model makes it particularly suited to the description of slow, repetitive activities such as walking. Accordingly, the force predictions obtained during walking have been well validated by comparison with values recorded from instrumented prostheses [9].

Whilst reliability and non-invasiveness are the principal advantages of the modelling technique, it also enables prediction of the effects of biomechanical manipulation for other system variables. For example, taking a subject’s pre-existing kinematic and kinetic dataset of level walking, it is possible to modify the objective function to reflect a greater activation of selected muscle groups and observe the consequences for the medial knee JRF over the course of the dynamic activity. Because input kinematic and kinetic data are constant between control and test conditions, this affords an opportunity to consider the effects of changes to muscular activation patterns in isolation.

It has been hypothesised that muscular factors drive joint pathology through the initiation and perpetuation of aberrant loading [6]. OA patients tend to suffer a number of muscular deficiencies, with loss of muscle strength out of proportion to loss of muscle cross-sectional area [10], a finding that illustrates the importance of factors beyond gross muscle structure in determining force generation. Muscle activation, a term that describes the coordinated neuromuscular process of aggregated myofibril recruitment leading to contraction, is one of these factors. Asked to contract the quadriceps maximally, patients with knee OA fail to achieve the same proportion of maximum force generation as do controls [11], suggesting that muscle force production in patients with OA might be increased by strategies targeting increased activation alone, without the need for increased muscle size. Decreased activation in patients has been attributed variously to pain inhibition, lack of motivation and neuromuscular dysfunction. With significant pathology of the central nervous system, as arises from stroke for example, muscular activation can be reduced to an extent that gives rise to frank weakness. Here, treatments that aim to increase activation, and thus power, must bypass the damaged central nervous system, and one way in which this can be achieved is by direct activation of muscle and nerve with electrical current, a technique that has widespread clinical use under the term functional electrical stimulation (FES) for the correction of foot drop following stroke [12]. Of course, the same technique may be used to increase activation in those without defined neurological dysfunction, allowing direct control of muscular power and timing, although this effect remains to be exploited clinically.

In the early stages of knee OA physiotherapy makes up a large part of treatment, comprising gait retraining regimes or strategies to increase muscular force generation, both of which may lead to alterations in muscular activation patterns [13]. These changes are made in the hope of favourable alterations to joint kinetics and resulting mitigation of pathological processes in articular cartilage. However, the optimal choice of muscle targets for inclusion in physiotherapy routines remains contentious. Traditionally, routines have focussed on those muscles at close proximity to the knee joint, but this is an approach that lacks a firm biomechanical basis; rather, a biomechanically sound analysis leads one to consider the hip musculature. As the controllers of frontal-plane pelvic motion the muscles around the hip, particularly gluteus medius, play a major role in stabilisation of the pelvis during gait [14]. Contraction of gluteus medius during stance phase limits contralateral pelvic drop; weakness of the muscle manifests as medial excursion of the bodily centre of mass as the pelvis drops towards the swing leg. To prevent instability this must be compensated, and this is achieved by shifting the torso in the opposite direction, towards the stance side, with each step, giving rise to the distinctive waddling motion known as the Trendelenburg gait, a clinical sign of gluteus medius weakness [15]. Replication of this increased trunk sway during gait in healthy individuals has been shown to reduce the EAM measured at the knee, confirming the importance of frontal plane motions of the central and upper body for the loads experienced more distally [16]. In patients with OA of the knee, pelvic kinetics have been shown to be of specific relevance to outcomes, with greater internal hip abduction moments during gait protecting against progression of disease from baseline to 18 months [17].

The results of clinical interventions specifically targeting the hip musculature have been mixed. In a large randomised controlled trial of hip muscle strengthening Bennell and colleagues [18] found no change in the EAM of patients with medial knee OA. Evidence for benefit is obtained from a more recent uncontrolled study by Thorp and colleagues investigating the use of intensive therapy directed towards gluteus medius in conjunction with more traditional quadriceps and hamstrings training [14]. Such a regime enabled subjects with OA of the knee to reduce the magnitude of the EAM by an average of 9 %. The discrepancy in outcomes might be accounted for by a greater emphasis on muscle activation in the latter study, where subjects were encouraged to learn the perceptions associated with contraction of gluteus medius. In Bennell’s study, hip abductor strength was indeed improved with training, but the authors acknowledged that the increased muscular capacity may not have been activated effectively during walking, an analysis supported by the observation that subjects showed *increased* pelvic drop following training. Pelvic drop is increasingly recognised as an important kinematic variable affecting loading at the knee. Thorp’s group posited a feasible biomechanical basis for the observed reduction in the EAM following training, hypothesising that decreased pelvic drop shifted the ground reaction force vector towards the stance leg (*lateralising* it), reducing the varus torque and thus the medial load acting at the knee.

The present study aimed to test this hypothesis. In the first instance, a virtual simulation of increased gluteus medius activation was performed in a normal walking dataset, through a manipulation of model optimisation parameters as described above. Such simulation allowed an analysis of the effects of increased activation in isolation without kinematic or ground kinetic change. FES was then used in a novel application to experimentally augment muscular activation, allowing an analysis of the full effects of changes to muscular activation in a real-world setting. Throughout, the tools of musculoskeletal modelling were used to determine internally acting forces.

## Methods

### Motion capture

Healthy subjects were sought for participation in the experimental protocol. Emails were sent to various mailing lists of Imperial College London, and posters were placed around the college campus.

The experimental setup for gait analysis comprised a set of ten Vicon optoelectronic cameras (Vicon MX system, Vicon Motion Systems Ltd, Oxford, UK) trained on a level walkway with a force plate (Kistler Type 9286AA, Kistler Instrumente AG, Winterthur, Switzerland) at its centre. Twelve single infrared reflective markers and two three-marker clusters were used to form a model of lower limb mechanics [8]. For each subject recorded data included a single static trial, where the subject stood motionless in neutral posture, and multiple dynamic trials. During the latter trials subjects walked normally across the walkway, taking several steps prior to landing with the right foot entirely on the force plate, and continuing for several steps thereafter; no instruction was given regarding walking speed. Between ten and 15 dynamic trials were recorded.

Following completion of motion capture of normal walking trials, the skin of the right gluteal region was prepared with 70 % isopropyl alcohol skin wipes and FES gel electrodes (PALS® Platinum, Axelgaard Manufacturing Co., Ltd, Fallbrook, CA, USA) were placed on the area overlying the right gluteus medius, along its line of action. The muscle was located by palpation, within the triangle formed by the right anterior superior iliac spine, right posterior superior iliac spine and the greater trochanter of the right femur. Gluteus maximus was avoided, as was the area superior to the iliac crest.

The electrodes were connected to a two-channel electrode stimulator (OCHS II, Odstock Medical Limited, Salisbury, UK) limited to a maximum current of 80 mA, with asymmetrical biphasic current waveforms of frequency 45 Hz. In order to check for effective electrode positioning the subject was asked to maintain left-legged stance while the stimulator was activated, with observation for ensuing abduction of the right leg to indicate contraction of gluteus medius. After confirming acceptability with the subject, the applied current was increased stepwise with actuation after each increase. The final stimulation current was chosen as that producing an abduction angle of 30–45 ° of the right leg whilst being tolerable. Subjects walked down the gangway with electrodes in situ, and FES was activated prior to right foot strike such that stimulation was maximal for the period of right stance. After several trial runs during which the subject became accustomed to the required timing, motion capture commenced. Again, between ten and 15 dynamic trials were recorded.

### Data processing

Data quality was contingent upon adequate marker visualization to allow reconstruction of relatively uninterrupted three-dimensional marker trajectories. Three control and three FES trials were taken for analysis from the lattermost recorded trials that fulfilled these requirements, to allow for adaptation to the imposed patterns of muscular activation. Initial processing was performed using Vicon Nexus® (1.85) and Matlab® (2015a; The MathWorks Inc., Natick, MA, USA). Data filtering was performed in Matlab using a low-pass fourth order Butterworth filter [19]. A cutoff frequency of 4 Hz was used on the basis of previous work showing that most of the frequency spectrum of the angular signals during walking lies below this threshold [20] and because the impact phase, in which the majority of the high-frequency information is contained, was not of primary interest. Filtering was uniformly applied to kinematic and kinetic data to prevent the introduction of artifacts resulting from incongruences between ground reaction force data and segment accelerations [21]. An open-source musculoskeletal model, Freebody (v1.1) [22], was used for subsequent data processing to determine internal forces. The model’s predictions of tibiofemoral JRF during gait have been validated using data from instrumented prostheses [23], and predicted muscle force waveforms have been shown to demonstrate high levels of concordance with known electromyography envelopes [22, 24]. The first part of the operation of Freebody involved the determination of coordinates of internal points (for example, bony landmarks and musculotendinous intersections) in a subject-specific frame of reference. This was achieved by scaling using the measurements of gender-matched subjects for whom three-dimensional position data of internal points were available, obtained using magnetic resonance imaging (method described in [23]). Processed data were then taken as input by a Matlab® implementation of optimisation using static trial data for model calibration, to determine muscle, joint and ligamentous forces for each sampled frame.

### Simulation of increased gluteus medius activation

Only normal walking trials were analysed for the purposes of simulation. Two separate optimisation routines were carried out for each trial: the first to reflect normal walking as the control condition, the second to simulate increased activation of gluteus medius through a manipulation of model parameters. For the former, the objective function used is described by:

1 $$ minimise\ J\kern0.5em  = {\sum}_{i\kern0.5em =\kern0.5em 1}^n{\left(\frac{F_i}{F_{imax}}\right)}^3; $$

for the latter:

2 $$ \begin{array}{l} minimise\ J = {\sum}_{i\kern0.5em =\kern0.5em 1}^nc.{\left(\frac{F_i}{F_{imax}}\right)}^3\kern0.75em \\ {}c = \left\{\begin{array}{c}\hfill 0.25\kern0.5em  gluteus\  medius\hfill \\ {}\hfill 1\  all\  other\  muscles\hfill \end{array}\right.\end{array} $$

where *F*_*i*_ is the force output of the *i* ^*th*^ muscle element, *F*_*imax*_ defines the *i* ^*th*^ muscle element’s force at maximum contraction and *n* is the total number of muscle elements (163) [25]. *F*_*imax*_ is calculated for each element from peak cross-sectional area, which is determined using subject-specific measurements and anatomical dataset values. The effect of discriminating between muscles by use of the variable weighting, *c*, is to alter the relative contributions of those muscles to the force-generating task defined by the input kinematic and kinetic data. Those muscles to which a lower value is attributed are ‘favoured’ by the model in driving a given movement because increasing their activation (increasing *F*_*i*_ in the above equations) contributes less to the objective function to be minimised, compared to increased activation of other muscles. With regards to the above equations, given the same kinematic and kinetic data, use of equation (2) results in a relatively greater activity apportioned to gluteus medius, with corresponding changes in other model variables including other muscular forces and joint reaction forces such that overall model constraints are satisfied. The value of 0.25 for *c* was chosen with the aim of inducing an increase in muscular force production corresponding roughly to the increase in maximum gluteus medius strength observed following training regimes in patients with knee OA, reported variously at 13, 19 and 50 % [14, 18], using data from a recent modelling study (unpublished observations, Xu R and Bull A).

Application of the two different optimisation routines to each trial consecutively produced paired model outputs for each normal walking trial of each subject, representing the control and FES-simulated conditions. This distinction provided the basis for comparison during subsequent data analysis.

### Experimental implementation of FES to gluteus medius

All trials (normal walking and FES) were taken for analysis. Freebody was used to calculate muscle and joint reaction forces by systematic application to data from each trial. For normal walking trials, the optimisation protocol employed equation (1) as the objective function. For FES trials, a modification was performed to account for the increased activation of gluteus medius, in a manner identical to that implemented in the preceding computer simulation, using equation (2) as the objective function. The distinction between normal walking trials and FES trials provided the basis for comparison during subsequent data analysis.

### Statistical methods

Statistical analyses were performed using Matlab® and applied consistently to data obtained from both the modelling and experimental studies. Time-integrated measures (impulses) were determined using the trapezoidal method of numerical integration [26], and all impulses were normalised to bodyweight to facilitate inter-subject comparison. Analysis of the medial knee JRF impulse was performed separately for mid-stance (17–50 % of stance) and terminal stance (51–83 %), and for the whole of stance phase. Early stance phase and the pre-swing phase were omitted in order to reduce the number of statistical tests performed and because these phases do not normally contain either of the two peaks of the stereotypical JRF-time curve within them. Components of the GRF were transformed into a local coordinate frame of reference defined by the evolving lower limb geometry in each frame, and impulses were calculated for these transformed forces. Thus the vertical component was defined collinear with the long axis of the shank, from the mid-point of the ankle to the mid-point of the tibial plateau. An intermediary plane containing this vector and that passing through the long axis of the foot, from mid-ankle to the head of the second metatarsal, was determined and used to calculate the anteroposterior component, defined as a vector orthogonal to the vertical component and lying within this plane. Finally, the mediolateral component was defined by a vector orthogonal to the vertical and anteroposterior components. In determining overall differences between conditions for joint reaction and muscular forces, GRF components and kinematic parameters, normality of the underlying distributions was assumed and two-way analyses of variance (ANOVAs) with repeated measures were performed. These tests took all individual un-averaged trial data into account (two conditions with three replications for each, per subject), with nominal variables given by subject and condition (condition defined as normal walking, FES-simulated or FES). Three different comparisons were made: normal walking versus FES-simulated, normal walking versus FES and FES-simulated versus FES. A significance threshold of *p* = 0.05 was applied throughout. For intervariable correlations coefficients of determination were calculated (presented as adjusted *R* ^*2*^ values) along with estimates and 95 % confidence intervals for beta coefficients, with *p* values determined by ANOVA.

## Results

Of the 16 healthy subjects who agreed to participate in the experimental protocol, one male subject was excluded on the basis of significant recent lower limb injury, leaving 15 who underwent testing (13 male, two female, age range 21–28, body mass index 21.6 ± 2.5 kgm^−2^). The range of stimulation currents administered to subjects varied from 31 mA to 80 mA (mean 52 mA). All participants tolerated FES well, and optimisation of both types was successful for all trials of all 15 subjects.

### Simulation of increased gluteus medius activation

**Fig. 1:**
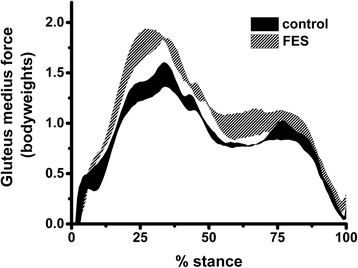
Mean and standard error of gluteus medius force across trials for one subject in normal walking and FES-simulated conditions. *FES* functional electrical stimulation

In simulations of increased gluteus medius activation, total impulse of the medial knee JRF was reduced by 4.2 % on average (*p* = 0.003) compared to normal walking, with a 6.5 % decrease in the magnitude of the mid-stance impulse (*p* = 0.001) and a 3.9 % decrease in the terminal-stance impulse (*p* = 0.070). Gluteus medius impulse was 33 % greater with FES simulation (*p* < 0.001) compared to normal walking (Fig. 1).

### Experimental implementation of FES to gluteus medius

#### Medial knee JRF

**Fig. 2:**
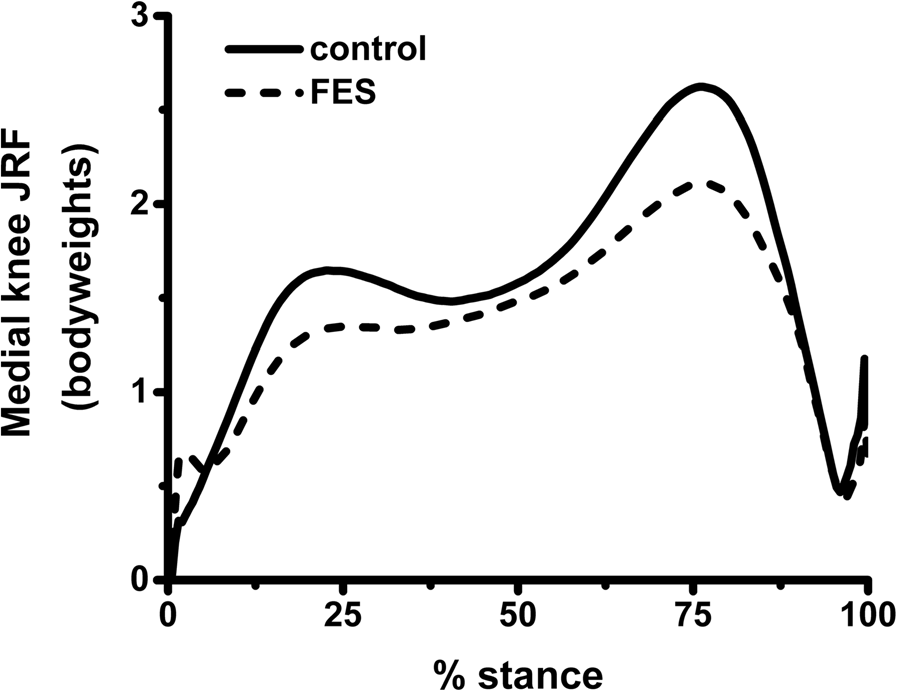
Average trajectory of the medial knee JRF across all subjects and all trials in normal walking and FES conditions. Individual trial data has been resampled to equate lengths. *FES* functional electrical stimulation, *JRF* joint reaction force

Thirteen of 15 subjects showed reductions in the medial knee JRF impulse in FES trials compared to normal walking. There were statistically significant decreases at mid-stance (*p* < 0.001), terminal stance (*p* < 0.001) and for the whole of stance (*p* < 0.001) when these phases were analysed independently. The average reduction in the total impulse across all subjects was 0.15 bodyweight-seconds, equivalent to a 12.5 % decrease from control; see Fig. 2. Mean reductions in peak force with FES were 13.8 % for the first peak and 18.4 % for the second peak of the medial knee JRF (*p* < 0.001 in each case) (Table 1).

**Table 1:** Mean and standard deviation of the medial knee JRF impulse (bodyweight-seconds) of each subject for analysed phases of stance, across normal walking, FES-simulated and FES trials

Subject	Medial knee JRF impulse (bodyweight-seconds)								
	Control			FES-simulated			FES		
	Mid-stance	Terminal stance	Total	Mid-stance	Terminal stance	Total	Mid-stance	Terminal stance	Total
1	0.40 ± 0.01	0.51 ± 0.01	1.13 ± 0.01	0.41 ± 0.01	0.51 ± 0.01	1.14 ± 0.01	0.36 ± 0.04	0.51 ± 0.03	1.10 ± 0.03
2	0.43 ± 0.05	0.45 ± 0.05	1.07 ± 0.11	0.40 ± 0.04	0.42 ± 0.05	1.01 ± 0.10	0.32 ± 0.03	0.33 ± 0.03	0.84 ± 0.12
3	0.42 ± 0.02	0.47 ± 0.02	1.16 ± 0.05	0.40 ± 0.02	0.46 ± 0.02	1.12 ± 0.04	0.33 ± 0.01	0.44 ± 0.03	0.96 ± 0.06
4	0.61 ± 0.03	0.69 ± 0.18	1.96 ± 0.21	0.56 ± 0.03	0.68 ± 0.18	1.88 ± 0.21	0.62 ± 0.03	0.68 ± 0.06	1.88 ± 0.13
5	0.41 ± 0.02	0.52 ± 0.01	1.16 ± 0.02	0.37 ± 0.02	0.51 ± 0.01	1.11 ± 0.02	0.40 ± 0.03	0.44 ± 0.04	1.05 ± 0.07
6	0.36 ± 0.05	0.92 ± 0.04	1.48 ± 0.03	0.35 ± 0.05	0.91 ± 0.05	1.47 ± 0.03	0.31 ± 0.05	0.63 ± 0.06	1.11 ± 0.06
7	0.41 ± 0.01	0.52 ± 0.01	1.15 ± 0.03	0.40 ± 0.01	0.52 ± 0.01	1.13 ± 0.04	0.41 ± 0.02	0.50 ± 0.04	1.12 ± 0.08
8	0.36 ± 0.02	0.53 ± 0.03	1.04 ± 0.03	0.36 ± 0.03	0.51 ± 0.03	1.02 ± 0.03	0.36 ± 0.01	0.53 ± 0.02	1.04 ± 0.03
9	0.21 ± 0.02	0.19 ± 0.01	0.52 ± 0.02	0.21 ± 0.02	0.19 ± 0.01	0.51 ± 0.03	0.23 ± 0.02	0.22 ± 0.03	0.59 ± 0.06
10	0.34 ± 0.04	0.56 ± 0.02	1.06 ± 0.04	0.27 ± 0.05	0.45 ± 0.08	0.88 ± 0.12	0.26 ± 0.05	0.43 ± 0.09	0.83 ± 0.13
11	0.34 ± 0.02	0.49 ± 0.04	1.04 ± 0.04	0.32 ± 0.02	0.44 ± 0.03	0.97 ± 0.03	0.18 ± 0.05	0.24 ± 0.04	0.56 ± 0.15
12	0.41 ± 0.07	0.58 ± 0.01	1.21 ± 0.08	0.37 ± 0.06	0.55 ± 0.01	1.13 ± 0.06	0.37 ± 0.06	0.57 ± 0.05	1.12 ± 0.14
13	0.53 ± 0.06	0.66 ± 0.02	1.45 ± 0.06	0.48 ± 0.05	0.62 ± 0.00	1.37 ± 0.06	0.62 ± 0.02	0.68 ± 0.03	1.57 ± 0.03
14	0.28 ± 0.01	0.52 ± 0.02	1.00 ± 0.03	0.26 ± 0.01	0.52 ± 0.02	0.98 ± 0.02	0.15 ± 0.01	0.28 ± 0.02	0.57 ± 0.01
15	0.33 ± 0.01	0.52 ± 0.01	1.07 ± 0.02	0.31 ± 0.01	0.52 ± 0.01	1.05 ± 0.03	0.29 ± 0.02	0.47 ± 0.01	0.99 ± 0.03

*JRF* joint reaction force, *FES* functional electrical stimulation

### Muscular forces

Mean gluteus medius impulse was 15 % greater in FES trials compared to control (*p* < 0.001).

### Kinematics

**Fig. 3:**
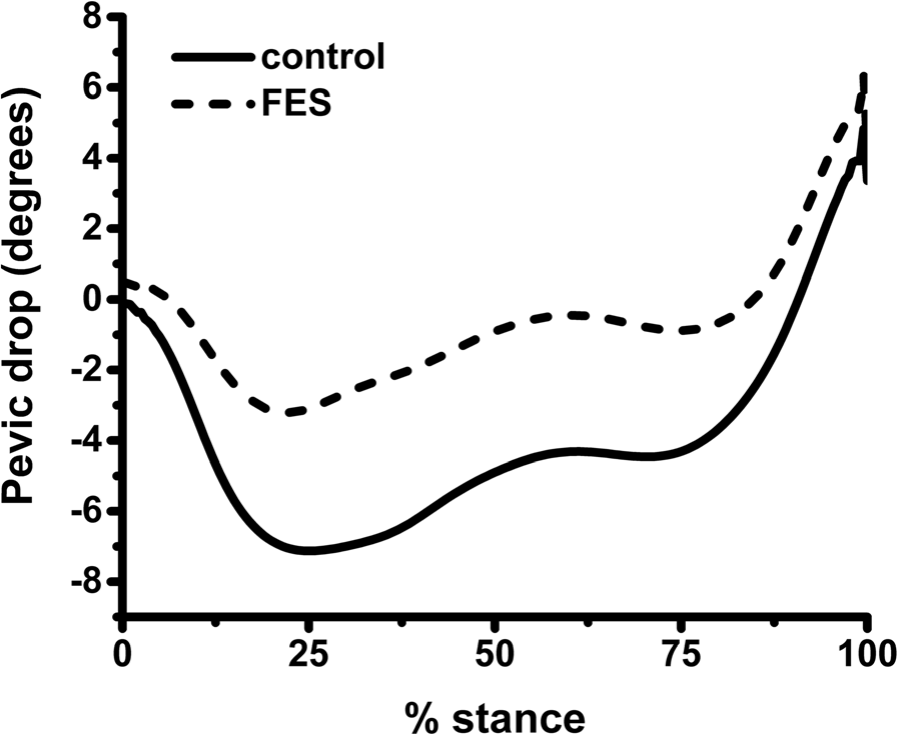
Average trajectory of pelvic drop angle in normal walking and FES conditions. Negative values indicate pelvic drop below horizontal. *FES* functional electrical stimulation

Peak excursions were compared for selected joint angles, revealing widespread kinematic change in FES trials compared to normal walking. Of particular significance were changes to the extent of pelvic drop in the frontal plane, towards the swing leg, during stance phase. In normal walking trials subjects tended to show some drop of the pelvis below the horizontal, peaking around 25 % of stance. With FES, the joint angle-time curve was up-shifted, with a 46 % reduction in peak pelvic drop (*p* < 0.001); see Fig. 3. Times to peak remained relatively unchanged.

Areas under the curve (AUCs) were computed for each joint angle-time curve by integrating across time, and averaging within condition across all subjects and all trials. FES resulted in significant changes in AUC for pelvic drop only (*p* = 0.002), which showed a large decrease. Averaging belied a significant degree of inter-subject variation in kinematic parameters. The average reduction in pelvic drop AUC with FES was plotted against the average reduction in the medial knee JRF impulse, for each subject. Strong positive correlation was observed; see Fig. 5a.

### Ground reaction force components

**Fig. 4:**
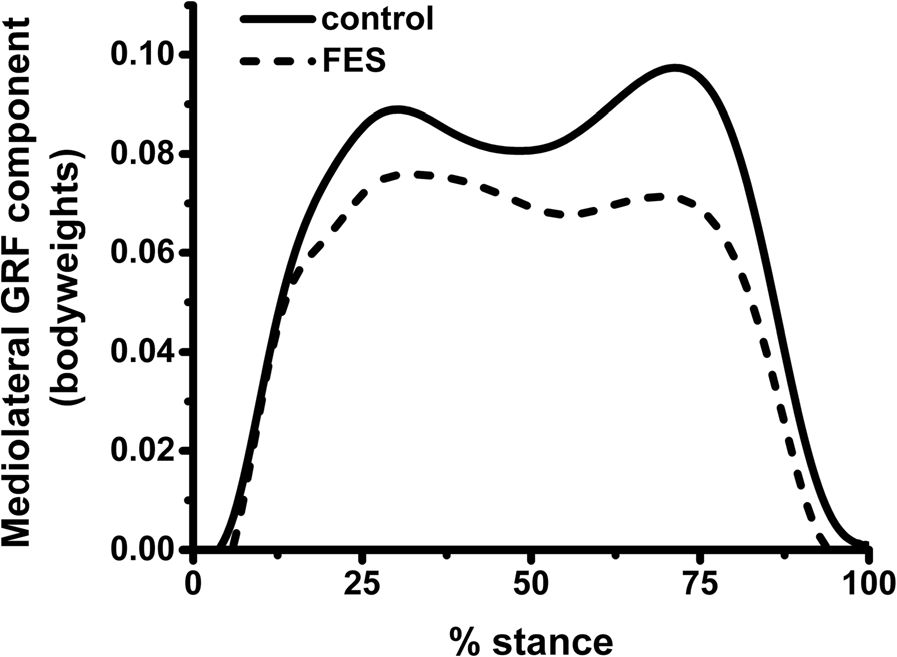
Average trajectory of mediolateral component of GRF in normal walking and FES conditions. *FES* functional electrical stimulation, *GRF* ground reaction force

The impulse of the mediolateral component of the GRF integrated across the whole of stance was reduced by 18 % in FES trials compared to normal walking (*p* < 0.001); see Fig. 4. In addition, there was a decrease in the vertical component impulse (*p* = 0.05) and an increase in the anteroposterior component impulse (*p* < 0.001).

**Fig. 5:**
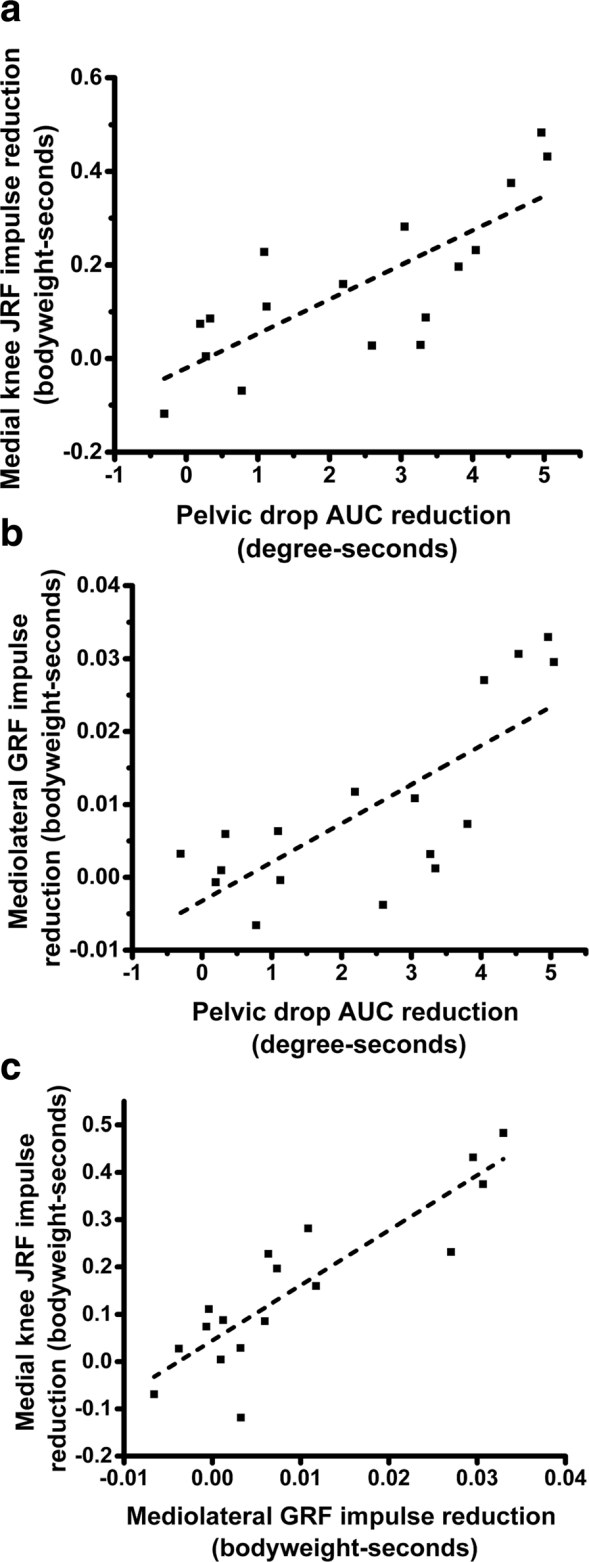
Intervariable correlations for change in pelvic drop, mediolateral component of GRF and medial knee JRF. Each point represents data from a single subject. For two subjects who received FES with two different current levels, results from both have been plotted. Strong positive correlation was found across all variables. **a**. Reduction in pelvic drop AUC versus reduction in medial knee JRF impulse (*R* = 0.78, *R* ^*2*^ = 0.59, *β* = 0.074, 95 % CI [0.041, 0.11], *p* < 0.001). **b**. Reduction in pelvic drop AUC versus reduction in mediolateral GRF impulse (*R* = 0.75, *R* ^*2*^ *=* 0.54, *β =* 0.0053, 95 % CI [0.0028, 0.0079], *p* < 0.001). **c**. Reduction in mediolateral GRF impulse versus reduction in medial knee JRF impulse (*R* = 0.88, *R* ^*2*^ = 0.75, *β* = 11.64, 95 % CI [8.11, 15.17], *p* < 0.001). *AUC* area under the curve, *GRF* ground reaction force, *JRF* joint reaction force

Significant inter-subject variation was observed in the degree of reduction of the mediolateral GRF component induced by FES. Plotting this reduction against the magnitude of reduction of pelvic drop AUC for individual subjects revealed strong positive correlation. There was also strong positive correlation with the magnitude of reduction of the medial knee JRF impulse; see Fig. 5b and c.

### Comparison of simulated and real increases in gluteus medius activation

FES-simulated trials were compared with real FES trials. While both resulted in reductions in the medial knee JRF impulse compared to control, reductions were significantly greater in real FES trials, where a further 8.6 % decrease was observed (*p* < 0.001).

## Discussion

The effects of specific muscular augmentation of gluteus medius on the medial knee JRF during level walking were investigated using motion capture and musculoskeletal modelling. Application of FES to gluteus medius during walking facilitated an average reduction in the medial knee JRF impulse of 12.5 % compared to non-stimulated trials. In a previous uncontrolled study it was hypothesised that reduced pelvic drop in the frontal plane might lead to a reduction in medial knee loading [14].

**Fig. 6:**
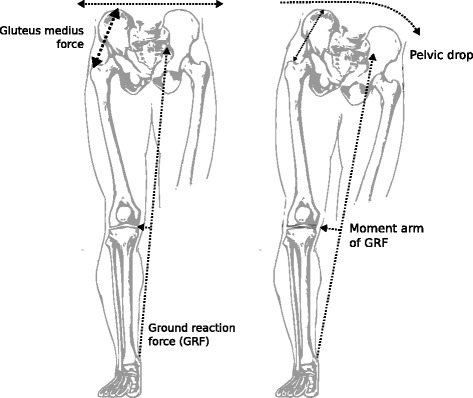
Schematic diagram showing how increased drop of the pelvis in the frontal plane leads to a lengthening of the moment arm of the GRF about the knee

A novel analysis of kinematics and kinetics was performed to test this hypothesis, ultimately lending weight to it. Crucially, across subjects, there were positive correlations between kinematic changes and changes to both the GRF and the medial knee JRF, with reductions in the medial knee JRF scaling with both the extent of reduction of pelvic drop and the degree of lateralisation of the GRF. Simulations of FES using a single dataset (thus neglecting kinematic effects) resulted in reductions in the medial knee JRF, but these were significantly lower than those obtained with real-world implementation of FES (where kinematic changes were often profound). The following explanation is proposed: FES activates gluteus medius during stance, which through increased contraction reduces the extent to which the pelvis drops towards the swing leg. This effect lateralises the bodily centre of mass (shifting it towards the stance leg) and in doing so lateralises the GRF vector. Lateralisation of the GRF reduces its moment arm about the knee and thus the resultant varus torque, in turn reducing the medial compressive force and so the medial knee JRF (Fig. 6).

The study’s significance lies in its confirmation of the central importance of proper gluteus medius function for the protection of the knee in established OA. This is something that remains to be fully reflected in physiotherapy regimes designed for patients, which often focus on those muscle groups at closer proximity to the joint. The results presented here support a primary role for gluteus medius rehabilitation in all such regimes. In addition, the study raises the possibility of intervention with FES to reduce the medial knee JRF in early OA. The mean values in reduction of peak medial knee JRF found here, of 13.8 % and 18.4 % for the first and second peak respectively, compare favourably with published reductions in the peak EAM following physiotherapy, where a mean reduction of 9 % was observed and patients reported large reductions in pain scores following intervention [14]. Whilst much of the previous work suggesting benefit from reduction of the medial knee JRF has focussed on peak values, many of the analyses of the results of the present study were performed using impulse. In the case of joint reaction force, this was partly to better approximate a marker of the disease-causing process. Studies in bovine cartilage support the existence of stress thresholds above which significant cellular damage begins to accumulate [27] but given the difficulty of applying a threshold in any meaningful manner in healthy subjects, whose knee JRFs, as measured by the EAM, tend to be much smaller in magnitude than those typically observed in OA patients [28], it was decided to use total area under the curve, or impulse, as the comparative metric. By taking account of time, this provided the added benefit of facilitating the intervariable correlations that were central to informing the study’s conclusions. In patients with knee OA the method has been validated to some extent by a study which showed that the external knee adduction impulse was more discriminative than peak knee adduction moment in predicting radiographic grade of knee OA [29]. The size of the proportional difference between the median values of the adduction impulse for patients in the moderate and mild OA groups was approximately 20 %, a value exceeded easily by the proportional reduction in the medial knee JRF impulse of five of the subjects tested in the present study.

### Validity of model predictions

Predicted model outputs were of an appropriate scale of magnitude, and JRF waveforms showed typical double-peaked shapes. The mean peak value for the medial knee JRF in control trials found in the present study of 2.6 bodyweights fits well within the scale of values found in other modelling studies and indeed shows no major discrepancy from those recorded in instrumented prostheses [30–33]. Significant inter-individual variation in the medial knee JRF was observed, as seen in patients; interestingly those subjects who showed the highest loads at baseline were among those who showed the greatest load reductions with FES.

Modification of the objective function was necessary to accurately model observed increases in the activation of gluteus medius in FES trials. By stimulating externally, the biological pathways through which muscle activation is regulated are bypassed, thus invalidating the use of optimisation in its standard form. The question was thus raised as to how best to reflect the increased contraction in the elements of gluteus medius and the interaction of this augmentation with the force outputs of other muscles. This is an open question. Whilst static optimisation has been verified to produce reasonable muscle force estimates in normal walking, its use in modelling FES-induced muscular contraction is a novel application. Ultimately, the value of 0.25 used for the constant, *c*, applied to gluteus medius activation was based on empirical observations regarding the extent of increased force produced using a range of different values. Temporal characteristics of muscle activation matched well with published data previously obtained during level walking [34] and the magnitudes of gluteus medius force obtained (around 1.5 to 2 bodyweights at peak) correspond roughly to those obtained elsewhere using musculoskeletal modelling (1 to 1.5 bodyweights), allowing for the fact that the latter figures are taken from a study into muscular forces in OA patients which are expected to be somewhat smaller [35]. Additional analysis of FES trials (not documented here) employing a standard objective function as per equation (1) demonstrated that the effect of altering gluteus medius force outputs on the medial knee JRF was relatively small compared to the overall effect, as apparent from closeness in the respective JRF-time curves derived using each objective function. In future, advances in electromyography and signal processing might provide a means by which to obtain quantitative data regarding the dynamics of muscle activation with FES. For now, taking all of the evidence together, the existence of a plausible biomechanical explanation for the obtained results, backed up strongly by the scaling of effects seen with the degree of change in pelvic drop and with the degree of lateralisation of the GRF, permits a high level of confidence in the accuracy of the model-predicted reductions in the medial knee JRF.

### Limitations

It remains to be seen if the effects generated here in young, healthy subjects can be replicated in patients with OA who differ from the test cohort in their morphology, bodily composition and kinematics [36, 37]. OA patients are predisposed to a number of muscular imbalances, including gluteus medius weakness [10]. A common problem with the use of FES is fatigue resulting from supraphysiological stimulation frequencies required to cause muscle activation and weakness can exacerbate this, limiting the potential for long-term stimulation. On the other hand, pre-existing muscular insufficiency might potentiate clinical effects achievable with FES. The widespread use of FES technology in foot drop shows that it can be successfully applied to elderly patients with comorbidities [12].

Use of FES may be limited by discomfort. Stimulation was on the whole well-tolerated, with none of the tested subjects complaining of more than moderate discomfort and all completing the entire experimental protocol. At 20–30 minutes testing with FES was brief, however, and may not be indicative of long-term tolerability. Moreover, there was significant variation in tolerated currents and the size of effects induced by stimulation at a given current. The latter might reflect differences in bodily composition at the stimulation site. Subjects with more subcutaneous tissue between skin and muscle are likely to require higher stimulating currents to obtain the same effect. OA patients are likely to carry more subcutaneous fat than subjects from the test cohort, potentially damping muscle activation.

Joint reaction forces at the lateral compartment of the knee, and at the hip, were analysed and demonstrated to be unchanged with FES. Though this is reassuring, effects of the kinematic change induced by FES on the integrity of other load-bearing structures need to be investigated further. Ultimately, the success of applying FES in patients will depend upon adequate beneficial effects obtainable within thresholds of discomfort and fatigue, and without excessive unwanted kinematic effects elsewhere. The finding of non-negligible effects on the medial knee JRF with simulation (and, therefore, in the absence of any kinematic change) may indicate that benefit can be obtained at low stimulation levels, with all the advantages that this entails in terms of tolerability and safety.

## Conclusions

The application of FES to gluteus medius during walking may facilitate significant reductions in the medial knee JRF of healthy subjects. This study provides a biomechanical rationale for the central importance of gluteus medius rehabilitation for patients with knee OA. In addition, the prospect of intervention with FES, to augment muscular contraction and slow disease progression, has been raised.

## Abbreviations

ANOVA: analysis of variance
AUC: area under the curve
EAM: external adduction moment
FES: functional electrical stimulation
GRF: ground reaction force
JRF: joint reaction force
OA: osteoarthritis
